# Estimated pulse wave velocity and dipstick proteinuria in young adults with MASLD: a cross-sectional health-check analysis

**DOI:** 10.3389/fmed.2026.1887276

**Published:** 2026-10-07

**Authors:** Chunxia Zhang, Jiaqing Yan, Ri Liu, Chen Qiu

**Affiliations:** 1 Ningbo Medical Center LiHuiLi Hospital (The Affiliated LiHuiLi Hospital of Ningbo University), Ningbo, Zhejiang, China; 2 The First Clinical Medical College, Zhengzhou University, Zhengzhou, Henan, China; 3 Chinese PLA General Hospital, Beijing, China

**Keywords:** arterial stiffness, dipstick proteinuria, dyslipidemia, estimated pulse wave velocity, metabolic dysfunction associated steatotic liver disease, urinary protein abnormalities

## Abstract

**Introduction:**

The estimated pulse wave velocity (ePWV) is a non—invasive surrogate measure of arterial stiffness that has been associated with cardiovascular risk and renal abnormalities. However, studies exploring the association between ePWV and dipstick proteinuria in patients with metabolic dysfunction—associated steatotic liver disease (MASLD)—a condition associated with an increased risk of chronic kidney disease (CKD)—are scarce. This study aimed to investigate the association between ePWV and dipstick proteinuria in patients with MASLD.

**Methods:**

This cross-sectional study included 1,641 MASLD patients from a cohort of individuals who underwent health check-ups at a tertiary hospital in Beijing, China. ePWV was calculated using an established equation based on age and mean arterial pressure (MAP). Participants were stratified based on ePWV tertiles. Multivariable logistic regression models were used to assess the relationship between ePWV and dipstick proteinuria, adjusting for potential confounders such as age, sex, BMI, and comorbid conditions. Subgroup analyses were performed according to sex, diabetes, hyperuricemia, dyslipidemia, *H. pylori* infection, and urolithiasis.

**Results:**

Unadjusted and adjusted models consistently showed that higher ePWV was associated with higher odds of dipstick proteinuria. In the highest ePWV tertile, the odds of dipstick proteinuria were higher compared to the lowest tertile (OR: 2.44, 95% CI: 1.20-5.67, *P* for trend = 0.002). Subgroup analysis revealed a statistically significant interaction by dyslipidemia status, with a stronger association observed among participants without dyslipidemia (OR: 4.59, 95% CI: 2.18-9.76, *P* = 0.001), while no statistically significant interactions were detected for sex, hyperuricemia, *H. pylori* infection, diabetes mellitus, or urolithiasis.

**Discussion:**

Higher ePWV was associated with an increased likelihood of dipstick proteinuria among patients with MASLD. Further longitudinal studies are needed to determine whether ePWV is associated with subsequent urinary protein abnormalities and kidney outcomes.

## 1 Introduction

Metabolic dysfunction–associated steatotic liver disease (MASLD), which clusters with hypertension, dyslipidemia, insulin resistance and chronic kidney disease (CKD) (1), has rapidly become the most common chronic liver disease globally. It is currently estimated to affect up to 38% of the global adult population and is expected to become the leading cause of chronic liver disease and a major indication for liver transplantation by 2030 (2, 3). A number of investigations have demonstrated that more severe phases of MASLD correlate with an increased frequency of chronic kidney disease (4). In a prospective investigation of 6,873 Chinese individuals, Liang found that ultrasound-confirmed MAFLD was independently linked to an elevated risk of developing CKD over a median follow-up of 4.6 years (adjusted HR 1.64; 95% CI 1.39–1.94) (5). Additionally, a separate cohort study encompassing 337,783 participants from the UK Biobank revealed that MAFLD was associated with an increased risk of incident ESRD (adjusted HR 2.03; 95% CI 1.68–2.46) (6).

Proteinuria is defined based on the Indicator dye–based dipstick test with a cut-off value of 1 + . Methods for detecting proteinuria include semi-quantitative dipstick testing and quantitative laboratory-based measurements. Quantitative measurement, especially immunoassay methods, have the advantage of high test accuracy, but the cost is high and requires the use of laboratory and electronic medical equipment, and is not suitable for large-scale screening (7). Urine dipstick testing is a low-cost and readily accessible method for detecting urinary protein abnormalities and may provide an indication of potential kidney involvement. However, dipstick results are semi-quantitative and may be influenced by various analytical and clinical factors; therefore, they should be interpreted within the appropriate clinical context (8).

Arterial stiffness reflects the cumulative effects of cardiometabolic risk factors and may increase pulsatile stress within the renal microcirculation (9). Carotid-femoral pulse wave velocity (cfPWV) is widely regarded as the reference non-invasive measure of arterial stiffness (10) and has been associated with cardiovascular events and mortality (11). However, widespread implementation of this technique remains constrained by requirements for dedicated devices and qualified operators (10). Therefore, our study uses an estimated PWV (ePWV) based on age and mean arterial pressure, which tracks vascular aging and associates with major outcomes, including heart failure, cognitive decline and cause-specific mortality (12–14). Research has shown that ePWV predicts all-cause and cardiovascular mortality in hypertensive populations (15). In the MORGAM Prospective Cohort Project involving 107,599 apparently healthy European individuals, elevated ePWV was associated with all-cause mortality, cardiovascular mortality, and composite cardiovascular endpoints after adjustment for SCORE and Framingham Risk Score, though its prognostic value was attenuated when adjusting for individual traditional cardiovascular risk factors (16). Similarly, analysis of the National Health and Nutrition Examination Survey (NHANES) 1999–2018 encompassing 18,956 US adults confirmed that ePWV remained associated with all-cause and cardiovascular mortality after adjustment for Framingham Risk Score and individual cardiovascular risk factors (HR: 1.22–1.35 per 1 m/s), with ePWV providing additional predictive value beyond traditional risk models (17). Beyond resting measurements, emerging evidence suggests that ePWV calculated from post-exercise blood pressure readings may offer superior prognostic information for all-cause mortality in young and middle-aged adults without cardiovascular disease, highlighting the potential for dynamic vascular assessment (18). Recent studies have also reported associations between elevated ePWV and adverse renal outcomes, suggesting that increased arterial stiffness may be related to renal hemodynamic stress (19, 20). But data specifically connecting ePWV with dipstick proteinuria in MASLD are scarce. Given MASLD's strong association with CKD and the practicality of dipstick testing in health-check settings, defining whether higher ePWV corresponds to greater odds of dipstick proteinuria could provide preliminary evidence for an association that may be related to renal microvascular and hemodynamic mechanisms (21, 22). Such evidence would also support the assessment of cardio-renal-metabolic risk by pairing an easily computed vascular index (ePWV) with an inexpensive renal readout (dipstick), highlighting a potential association that may inform future hypothesis-driven research. This study therefore evaluates the association between ePWV and dipstick proteinuria among adults with MASLD undergoing standardized health examinations.

Against this background, the present study aimed to investigate the cross-sectional association between estimated pulse wave velocity (ePWV) and dipstick proteinuria in a cohort of patients with metabolic dysfunction-associated steatotic liver disease (MASLD). Specifically, the primary exposure was ePWV, examined both as a continuous variable and across tertile-based categories. The primary outcome was dipstick proteinuria, defined as a urinalysis result of ≥ 1 + . Secondary analyses included stratified subgroup examinations to explore potential effect modifications by sex, type 2 diabetes mellitus (T2DM), hyperuricemia, dyslipidemia, urolithiasis, and *H. pylori* infection. We hypothesized that higher ePWV levels would be associated with an increased likelihood of dipstick proteinuria in this population; however, we emphasize that this hypothesis pertains to an association within the selected MASLD population rather than a causal pathway, as the study design does not permit causal inference regarding the role of MASLD as a mediator or effect modifier.

## 2 Methods

### 2.1 Study design and setting

We performed a single-center, cross-sectional analysis of adults who attended routine comprehensive health check-ups at a tertiary hospital in Beijing, China. The study population comprised participants with hepatic steatosis identified by abdominal ultrasonography and at least one cardiometabolic risk factor, thereby meeting the prespecified metabolic and imaging criteria used to identify the MASLD population in this study. Alcohol consumption was assessed during the health examination; however, information on alcohol intake was unavailable for most participants and therefore could not be incorporated into the primary analyses. Consequently, alcohol-related steatotic liver disease or mixed etiologies could not be completely excluded and may have resulted in potential misclassification of the MASLD population (1, 21, 22). Given the *a priori* interest in arterial stiffness and urinary protein abnormalities in a relatively young population, we limited the analysis to participants aged 18–39 years. This predefined age range was selected to focus on the association between ePWV and dipstick proteinuria in young adults with MASLD. Furthermore, identifying urinary protein abnormalities in young adults with MASLD may help inform future studies of early kidney involvement and subsequent kidney outcomes. Arterial stiffness was assessed using ePWV, an estimated surrogate measure of arterial stiffness derived from age and mean arterial pressure (MAP) using a validated reference equation (9, 12, 14).

### 2.2 Patient selection

From 13,415 examinees, we selected young adults aged 18 to 39 as our initial screening target. We then excluded those without mean blood pressure data, and those without dipstick proteinuria results. Following the application of exclusion criteria, a total of 5,435 participants received a diagnosis of steatotic liver disease (SLD) based on abdominal ultrasound findings. Furthermore, eligibility required that each participant satisfy at least one of the established criteria for cardiometabolic risk factors: (1) BMI ≥ 23 kg/m^2^ or waist circumference > 90 cm in men or > 80 cm in women. (2) Fasting blood glucose ≥ 100 mg/dL (5.6 mmol/L) or 2-hr post-glucose load ≥ 140 mg/dL (7.8 mmol/L) or HbA1c ≥ 5.7% or on specific drug treatment. (3) Blood pressure ≥ 130/85 mmHg or on specific drug treatment; (4) Plasma triglycerides ≥ 150 mg/dL (1.70 mmol/L); (5) Plasma HDL cholesterol < 40 mg/dL (1.0 mmol/L) for men and < 50 mg/dL (1.3 mmol/L) for women or on specific drug treatment (3). The final analytic sample comprised 1,641 participants who met the prespecified hepatic steatosis and cardiometabolic criteria for the MASLD study population, of whom 118 had dipstick proteinuria and 1,523 did not. For exposure categorization, ePWV was divided into tertiles: 4.96 **≤** ePWV < 6.57, 6.57 **≤** ePWV < 7.13, and 7.13 ≤ ePWV ≤ 9.71.

### 2.3 Definitions

Dipstick proteinuria. Urine protein was measured from a spot mid-stream sample using AVE-11 series dipsticks (Ave Science & Technology Co., Ltd., Hunan, China) processed by an AVE-75 series automatic urine analyzer at the health-check center. The dipstick test is based on the tetrabromphenol blue protein-error-of-indicator principle, which predominantly detects albumin rather than total protein. According to the manufacturer's specifications, the approximate protein concentrations corresponding to dipstick grades are: trace (∼10–20 mg/dL), 1+ (∼30 mg/dL), 2+ (∼100 mg/dL), 3+ (∼300 mg/dL), and 4+ (∼1000 mg/dL). Consistent with epidemiologic practice and to enhance specificity, dipstick proteinuria was defined as ≥ 1 + on the ordinal scale, recognizing the test's convenience and known analytical limitations (8, 23).

Estimated PWV. ePWV was used as an estimated measure of arterial stiffness and calculated from age and MAP using the validated reference equation derived against cf-PWV (11, 24). With age in years and MAP in mmHg, the formula was:

$$\mathrm{ePWV}\;(m/s)=9.587\;\;-\;\;0.402\times \mathrm{age}\;\;+\;\;4.560\times 10^{-3}\times \mathrm{ag}e^{2}\;\;-\;\;2.621\times 10^{-5}\times \mathrm{ag}e^{2}\times \mathrm{MAP}\;\;+\;\;3.176\times 10^{-3}\times \mathrm{age}\times \mathrm{MAP}\;\;-\;\;1.832\times 10^{-2}\times \mathrm{MAP}.$$

MAP was computed as DBP + 0.4 × (SBP−DBP), as recommended in prior ePWV applications to health-check and ICU cohorts (20). Higher ePWV values are generally interpreted as reflecting greater arterial stiffness, and have shown prognostic value across cardiovascular and renal outcomes (11, 12). In the present study, ePWV was analyzed continuously and by tertiles (4.96–6.57, 6.57–7.13, and 7.13–9.71 m/s).

### 2.4 Data collection and covariates

Data collected during routine medical check-ups included demographic information (age, sex, body mass index [BMI], waist circumference [WC] and vital signs [SBP, DBP]), clinical history (hypertension [HTN], type 2 diabetes mellitus [T2DM], dyslipidemia, hyperuricemia, urolithiasis, and Helicobacter pylori [*H. pylori*] infection), and laboratory test results. HTN was determined when an individual presented with a systolic blood pressure of 140 mmHg or higher, a diastolic blood pressure of 90 mmHg or higher, or was currently taking antihypertensive agents (25). T2DM was diagnosed according to the following criteria: a fasting plasma glucose level of at least 126 mg/dL, a two-hour plasma glucose reading of 200 mg/dL or greater during an oral glucose tolerance test, or the use of glucose-lowering medications (26). Dyslipidemia was diagnosed if any of the following conditions were met: a total cholesterol (TC) level of 5.18 mmol/L or above, a low-density lipoprotein cholesterol (LDL-C) level of 3.37 mmol/L or above, a triglyceride (TG) level of 1.7 mmol/L or above, or the current use of lipid-lowering therapy (27). Hyperuricemia was defined as serum uric acid (SUA) > 420 μmol/L (28). The diagnosis of urolithiasis was based on patient self-reports, while *H. pylori* infection was identified through the urea breath test (29). Alcohol consumption was queried during the health check-up. However, due to a high proportion of missing responses, it was not included as a covariate in the primary regression models.

### 2.5 Statistical modeling

To estimate the association between ePWV and dipstick proteinuria, we used logistic regression across: (i) an unadjusted model; (ii) Model 1 adjusted for sex, age, BMI, and WC; (iii) Model 2 further adjusted for SCr, BUN, Lp(a), and WBC; and (iv) Model 3 additionally adjusted for HTN, T2DM, dyslipidemia, urolithiasis, and *H. pylori* infection. Odds ratios (ORs) with 95% confidence intervals (CIs) were reported for ePWV as a continuous variable and by tertiles, using the lowest tertile as reference, consistent with the structure of Table 2 and the subgroup framework in Figure 2. To assess the robustness of the primary findings to the choice of inflammatory marker, sensitivity analyses were performed by separately replacing WBC with NEU, MON, LYM, or CRP in the fully adjusted Model 3. Each inflammatory marker was evaluated in a separate model, while all other covariates remained identical to those in Model 3. Thus, the inflammatory markers were not entered simultaneously into the same model to minimize potential multicollinearity among correlated inflammatory markers (Supplementary Table S2). Model diagnostics were performed for the fully adjusted model (Model 3) and each sensitivity model. Multicollinearity was assessed using variance inflation factor (VIF), with a VIF > 5 considered indicative of substantial collinearity (Supplementary Table S1). To flexibly model the association between ePWV (as a continuous variable) and dipstick proteinuria, we performed restricted cubic spline (RCS) regression with 4 knots (placed at the 5th, 35th, 65th, and 95th percentiles of the ePWV distribution) in the fully adjusted model. Nonlinearity was tested using the likelihood ratio test comparing the model with only the linear term against the model with linear and spline terms (Supplementary Figure S2).

## 3 Results

### 3.1 Participant selection and baseline characteristics

A total of 13,415 participants who underwent comprehensive health check-ups were initially assessed, and after applying exclusion criteria, 1,641 individuals were included for the final analysis (Figure 1). The baseline demographic and clinical characteristics of the study population, categorized by tertiles of ePWV, showed significant differences across the groups. Participants in Tertile 3 (ePWV ≥ 7.13 m/s) were older (33.0 ± 4.4 years) compared to those in Tertile 1 (30.4 ± 4.7 years, *P* < 0.001; Table 1). Additionally, BMI and WC were higher in Tertile 3, indicating increased central obesity (*P* < 0.001 for both; Table 1). A higher proportion of males was observed in Tertile 3 (92.9%, *P* < 0.001; Table 1). A significant association with metabolic and inflammatory markers was also found, with WBC, NEU, and MON all being higher in Tertile 3 (*P* < 0.001 for all three; Table 1). The prevalence of HTN increased with higher ePWV tertiles, reaching 6.8% in Tertile 3 (*P* < 0.001; Table 1). Similarly, T2DM prevalence was higher in Tertile 3 (11.2%, *P* < 0.001; Table 1), and dyslipidemia was more common in the higher tertiles (*P* < 0.001; Table 1). Participants in Tertile 3 also had significantly higher prevalence of hyperuricemia (HUA) (*P* < 0.001; Table 1) and a higher rate of *H. pylori* infection (*P* = 0.038; Table 1). Although substantial overlap existed between the distribution of ePWV stratified by dipstick proteinuria status, participants with dipstick proteinuria showed an upward shift in the ePWV distribution compared to those without dipstick proteinuria (Supplementary Figure S1).

**Figure 1:**
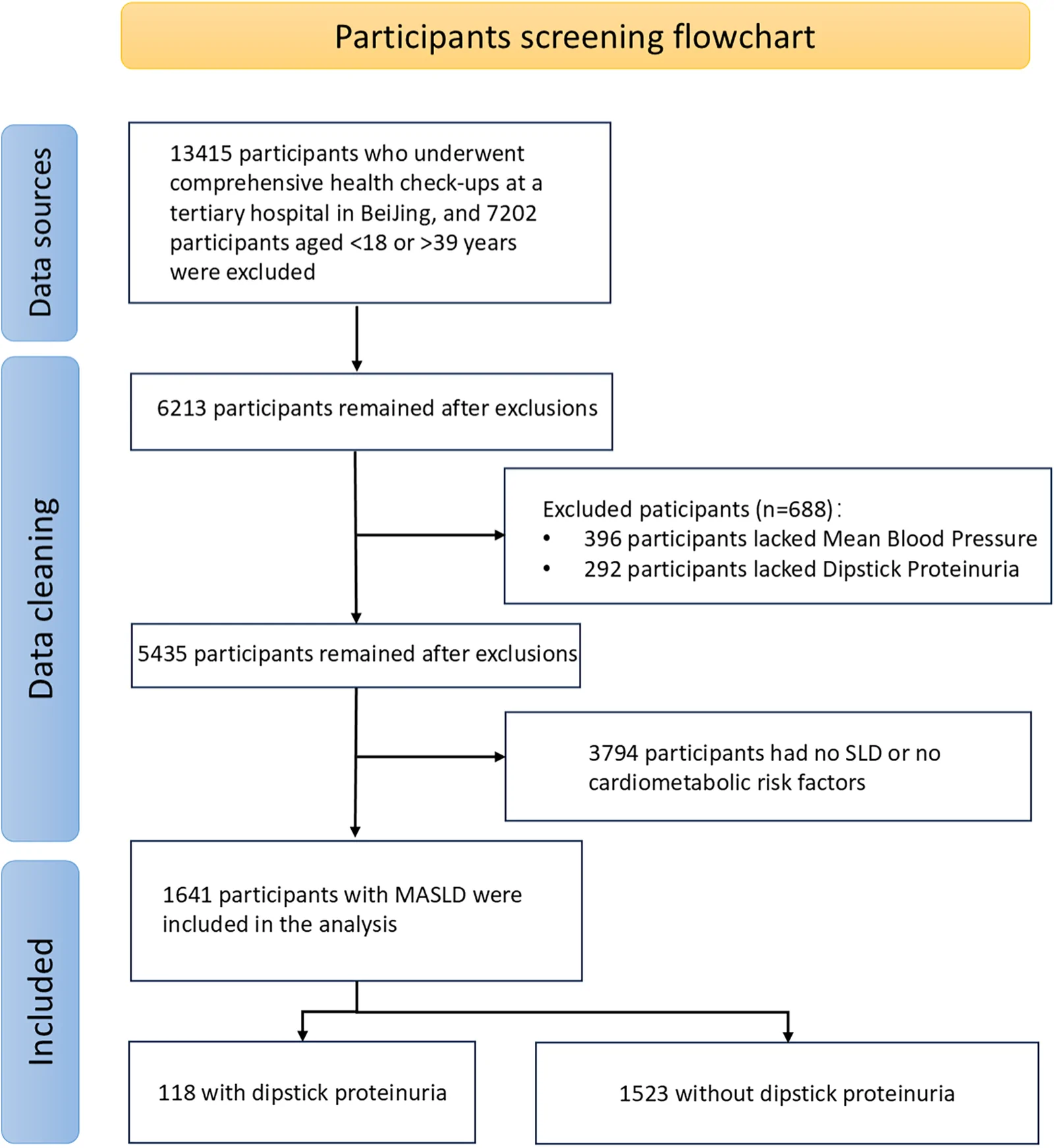
Flowchart of participant selection.

**Table 1:** Baseline characteristics of the study population according to ePWV tertiles

Variables	Total	ePWV			*P*	*SMD*	
		T1	T2	T3		T2 vs. T1	T3 vs. T1
Participants	1,641	547	547	547			
Sex, *n* (%)					<0.001	0.239	0.399
Male	1,424 (86.8)	434 (79.3)	482 (88.1)	508 (92.9)			
Female	217 (13.2)	113 (20.7)	65 (11.9)	39 (7.1)			
Age, years	31.5 ± 4.8	30.4 ± 4.7	31.0 ± 4.8	33.0 ± 4.4	<0.001	0.134	0.582
BMI, kg/m^2^	27.2 ± 3.5	26.1 ± 2.8	27.2 ± 3.4	28.3 ± 4.0	<0.001	0.373	0.641
WC, cm	93.5 ± 9.5	90.3 ± 8.4	93.9 ± 9.5	96.2 ± 9.8	<0.001	0.391	0.638
SCr, μmol/L	71.6 ± 12.9	70.3 ± 13.6	72.1 ± 12.7	72.3 ± 12.4	0.015	0.140	0.159
BUN, mmol/L	4.9 ± 1.1	4.8 ± 1.1	4.9 ± 1.0	4.9 ± 1.1	0.475	0.069	0.013
Lp(a), g/L	0.1 (0.0, 0.2)	0.1 (0.0, 0.2)	0.1 (0.0, 0.2)	0.1 (0.0, 0.3)	0.167	0.123	0.194
WBC, ×10^9^/L	6.8 ± 1.6	6.6 ± 1.5	6.8 ± 1.6	7.0 ± 1.8	<0.001	0.176	0.256
NEU, ×10^9^/L	3.9 ± 1.2	3.8 ± 1.2	3.9 ± 1.1	4.0 ± 1.3	<0.001	0.146	0.232
MON, ×10^9^/L	0.4 ± 0.1	0.4 ± 0.1	0.4 ± 0.1	0.4 ± 0.1	<0.001	0.257	0.273
LYM, ×10^9^/L	2.3 ± 0.6	2.2 ± 0.6	2.3 ± 0.7	2.3 ± 0.6	0.025	0.108	0.169
CRP, mg/L	3.0 (0.5, 3.0)	3.0 (0.5, 3.0)	3.0 (0.5, 3.0)	3.0 (0.5, 3.0)	0.989	0.024	0.050
HTN, *n* (%)	46 (2.8)	1 (0.2)	8 (1.5)	37 (6.8)	<0.001	0.142	0.365
T2DM, *n* (%)	113 (6.9)	15 (2.7)	37 (6.8)	61 (11.2)	<0.001	0.190	0.335
Urolithiasis, *n* (%)	157 (9.6)	48 (8.8)	49 (9)	60 (11)	0.392	0.006	0.074
HUA, *n* (%)	912 (55.6)	267 (48.8)	322 (58.9)	323 (59)	<0.001	0.201	0.206
Dyslipidemia, *n* (%)	1,167 (71.1)	355 (64.9)	377 (68.9)	435 (79.5)	<0.001	0.086	0.331
*H.pylori* infection, *n* (%)	403 (24.6)	131 (23.9)	118 (21.6)	154 (28.2)	0.038	0.057	0.096

Data are presented as mean ± SD, median (IQR), or *n* (%). ePWV, estimated pulse wave velocity; BMI, body mass index; WC, waist circumference; SCr, serum creatinine; BUN, blood urea nitrogen; Lp(a), lipoprotein(a); WBC, white blood cell count; NEU, neutrophil count; MON, monocyte count; LYM, lymphocyte count; CRP, C-reactive protein; HTN, hypertension; T2DM, type 2 diabetes mellitus; HUA, hyperuricemia; *H. pylori* infection, Helicobacter pylori infection. SMD, standardized mean difference. *P* values represent overall comparisons across the three ePWV tertiles. SMDs were calculated using the lowest ePWV tertile (T1) as the reference group.

### 3.2 Associations between ePWV and dipstick proteinuria

To evaluate the association between ePWV and dipstick proteinuria among individuals with MASLD, we performed multivariable logistic regression analyses. In the unadjusted model, ePWV was significantly associated with dipstick proteinuria, with an odds ratio (OR) of 1.70 (95% CI: 1.32–2.18, *P* < 0.001) for the entire study population (Table 2). As ePWV increased, the association became more pronounced in higher tertiles, with ORs of 1.73 (95% CI: 1.01–2.98) in Tertile 2 and 2.89 (95% CI: 1.74–4.78) in Tertile 3 (Table 2).

**Table 2:** Association between ePWV and dipstick proteinuria in MASLD patients.

Variable	ePWV (per 1 m/s increase) (*n* = 1,641)		ePWV			*P* for trend
			4.96 ≤ ePWV<6.57 (*n* = 547)	6.57 ≤ ePWV < 7.13 (*n* = 547)	7.13 ≤ ePWV≤9.71 (*n* = 547)	
	OR (95% CI)	*P* value	OR (95% CI)	OR (95% CI)	OR (95% CI)	
Unadjusted	1.70 (1.32–2.18)	<0.001	1 (Ref)	1.73 (1.01–2.98)	2.89 (1.74–4.78)	<0.001
Model 1	1.65 (1.24–2.19)	0.001	1 (Ref)	1.63 (0.94–2.83)	2.70 (1.57–4.65)	<0.001
Model 2	1.65 (1.23–2.20)	0.001	1 (Ref)	1.23 (0.56–2.99)	2.49 (1.23–5.78)	<0.001
Model 3	1.61 (1.20–2.16)	0.001	1 (Ref)	1.22 (0.55–2.99)	2.44 (1.20–5.67)	0.002

Data are presented as odds ratios (OR) with 95% confidence intervals (CI). Model 1 adjusted for sex, age, BMI, and WC. Model 2 further adjusted for SCr, BUN, Lp(a), WBC. Model 3 additionally adjusted for HTN, T2DM, dyslipidemia, urolithiasis, and *H. pylori* infection. ePWV, estimated pulse wave velocity; MASLD, metabolic dysfunction-associated steatotic liver disease; other abbreviations as in Table 1.

After adjusting for basic factors such as sex, age, BMI, and WC (Model 1), the association remained significant, with an OR of 1.65 (95% CI: 1.24–2.19, *P* = 0.001; Table 2) for the total population. In Tertile 3, the OR was 2.70 (95% CI: 1.57–4.65), compared with the lowest tertile, and the *P* for trend was <0.001 (Table 2), suggesting a stronger relationship between elevated ePWV and dipstick proteinuria. The association persisted after further adjustment for clinical variables, including SCr, BUN, WBC, and comorbidities such as HTN and T2DM (Model 2 and Model 3), with ORs remaining statistically significant in the highest tertile, but not in the middle tertile (Table 2). Sensitivity analyses using alternative inflammatory markers yielded results consistent with those of the primary analysis. Specifically, WBC was separately replaced by NEU, MON, LYM, or CRP in the fully adjusted model, and the positive association between ePWV and dipstick proteinuria remained materially unchanged across these models (Supplementary Table S2). Restricted cubic spline analysis with 4 knots suggested a significant nonlinear relationship between ePWV and dipstick proteinuria (*P* for nonlinearity = 0.009, Supplementary Figure S2), indicating that the association is not strictly linear across the full range of ePWV values. These findings indicate that the association was not adequately described by a single linear term across the observed ePWV range.

### 3.3 Subgroup analysis

To evaluate the consistency of the association between ePWV and dipstick proteinuria across different clinical subgroups, we conducted stratified analyses by sex, T2DM, HUA, dyslipidemia, urolithiasis, and *H. pylori* infection (Figure 2). After full adjustment for confounders, the positive association between ePWV and dipstick proteinuria remained consistent across most subgroups, with no significant interactions observed for sex, T2DM, HUA, urolithiasis, or *H. pylori* infection (all *P* for interaction > 0.05). However, a statistically significant interaction was detected for dyslipidemia (*P* for interaction = 0.001). Specifically, among participants without dyslipidemia, higher ePWV was strongly associated with increased odds of proteinuria (OR: 4.59, 95% CI: 2.18–9.76), whereas no significant association was observed in those with dyslipidemia (OR: 1.31, 95% CI: 0.94–1.82).

**Figure 2:**
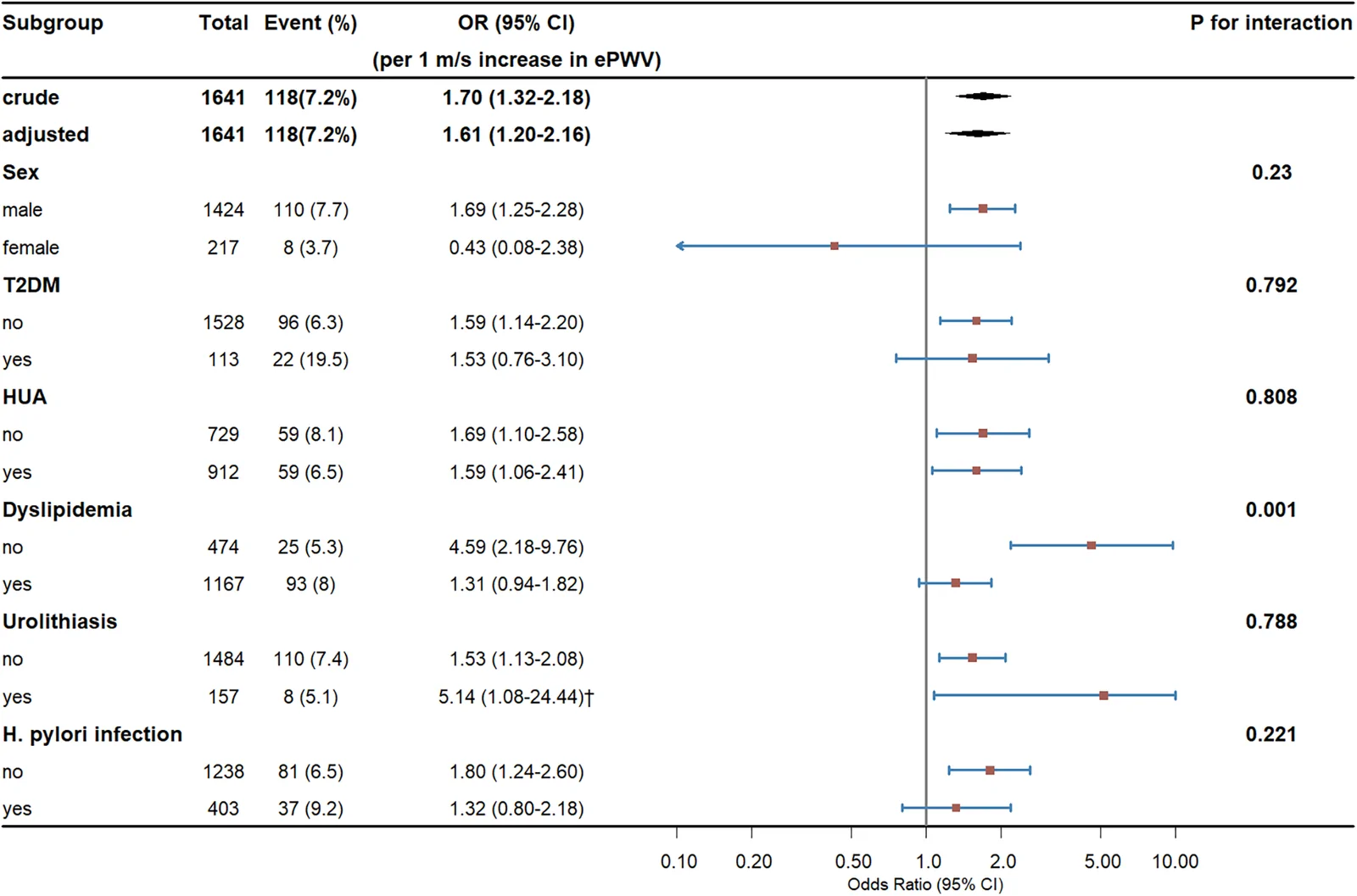
Subgroup analysis of the association between ePWV and dipstick proteinuria. Odds ratios (ORs) and 95% confidence intervals (CIs) were estimated using multivariable logistic regression models adjusted for the covariates included in Model 3 (Table 2), except for the variable defining the corresponding subgroup. Subgroups were defined by sex, type 2 diabetes mellitus (T2DM), hyperuricemia (HUA), dyslipidemia, urolithiasis, and H. pylori infection. *P* values for interaction are shown. ORs represent the change in odds of dipstick proteinuria per 1 m/s increase in ePWV. †: Upper confidence interval truncated at 10.

## 4 Discussion

### 4.1 Principal findings

In this cross-sectional cohort of 1,641 adults with MASLD, greater arterial stiffness—indexed by ePWV—was consistently associated with higher odds of dipstick proteinuria. In unadjusted analyses, each 1 m/s increase in ePWV was associated with higher odds of dipstick proteinuria, and this association remained statistically significant after sequential adjustment for demographics and adiposity, kidney and inflammatory biomarkers, and cardiometabolic comorbidities. When ePWV was categorized, the highest tertile (7.13–9.71 m/s) showed higher odds of dipstick proteinuria than the lowest tertile (4.96–6.57 m/s) in both crude and fully adjusted models, whereas the middle tertile showed a smaller, statistically non-significant association after full adjustment (Table 2). Baseline profiles were also characterized by increasing levels of cardiometabolic risk factors across ePWV tertiles, as participants in the highest tertile were older, more centrally obese, and had a higher burden of HTN, T2DM, dyslipidemia, and hyperuricemia (all *P* < 0.05; Table 1). Notably, this association persisted in subgroups stratified by sex, hyperuricemia, *H. pylori* infection, and urinary tract stones, suggesting that the observed association was broadly consistent across these subgroups. However, a statistically significant interaction was observed according to dyslipidemia status (*P* for interaction = 0.001; Figure 2). This subgroup finding was unexpected and should be interpreted with caution. Several factors may have contributed to this observation, including residual confounding, treatment effects such as statin use, multiple testing, or the relatively small number of events in the non-dyslipidemia subgroup, as reflected by the wide confidence interval (OR: 4.59, 95% CI: 2.18–9.76). Given these uncertainties, this finding should be considered exploratory and hypothesis-generating rather than conclusive, and confirmation in larger prospective studies is required.

### 4.2 Mechanistic interpretation

Biologically, arterial stiffening has been proposed to contribute to renal microvascular dysfunction through enhanced transmission of pulsatile energy, impaired microvascular reactivity, and structural remodeling of the renal vasculature (15, 30). Accumulating evidence suggests a bidirectional relationship between vascular dysfunction and kidney disease, in which hemodynamic disturbances and structural alterations in large arteries may contribute to the development and progression of CKD. In the Age, Gene/Environment Susceptibility-Reykjavik Study, higher aortic stiffness was associated with increased pulsatility of flow in the renal arteries, suggesting that excessive flow pulsatility may be transmitted into the renal microcirculation, which is particularly susceptible to hemodynamic stress because of its high resting blood flow and low vascular resistance (31, 32). Such hemodynamic stress may contribute to microvascular and glomerular abnormalities (31, 33). Collectively, these findings suggest that aortic stiffening may alter the transmission and reflection of pulsatile energy within the renal circulation, potentially increasing pulsatile stress in the renal microvasculature (34, 35). These proposed mechanisms provide a potential biological basis for the observed association between ePWV and dipstick proteinuria, although the present cross-sectional study cannot establish causality or determine whether the observed dipstick proteinuria represents persistent urinary protein abnormalities.

### 4.3 Comparison with existing literature

Our outcomes correspond to and extend a growing body of work linking ePWV to adverse cardio-renal outcomes. Prior cohorts have reported associations between higher ePWV and incident heart failure (12), peri-procedural acute kidney injury after coronary revascularization (20), and cause-specific mortality (14), supporting the broader association between ePWV and clinically relevant cardiovascular and kidney-related outcomes. In metabolically unhealthy populations, recent data from U.S. young adults demonstrated a positive, approximately linear association between ePWV and MASLD itself (36), consistent with the higher cardiometabolic risk burden observed at higher ePWV tertiles, including greater adiposity and a higher prevalence of hypertension, diabetes, and dyslipidemia.

### 4.4 Methodological considerations and key findings

Unlike most prior studies that focused on “hard” outcomes (HF, AKI, death) (12, 14, 20), we examined dipstick proteinuria as a low-cost and readily available measure of urinary protein abnormalities that is widely used in routine clinical practice. Mechanistically, our observations accord with contemporary frameworks in which large-artery stiffening amplifies pulsatile load to the renal microcirculation, potentially contributing to increased urinary protein excretion (37, 38). Methodologically, we used the validated reference ePWV equation derived against cf-PWV (24), similar to prior epidemiologic work (12, 15). A notable difference is the stronger association we observed among participants without dyslipidemia. Collectively, our data bridge the literature on ePWV and urinary protein abnormalities in MASLD by demonstrating a nonlinear association between ePWV and dipstick proteinuria in a relatively young, health-check cohort, complementing and expanding the scope of recent ePWV studies.

A distinctive feature of this study is the integration of a standardized health-check workflow—synchronous blood pressure measurement, urinalysis, anthropometrics, and laboratory panels within a single visit—allowing precise alignment of ePWV measurements with dipstick proteinuria assessment in a relatively young MASLD cohort (18–39 years) and minimizing potential temporal misclassification (8, 23). We operationalized arterial stiffness using the validated reference ePWV equation derived against cf-PWV and computed mean arterial pressure as DBP + 0.4 × (SBP–DBP), an approach consistent with recent cardio-renal applications of ePWV (15, 20, 24). Methodologically, we implemented a prespecified tiered adjustment strategy that first controlled for demographics and adiposity, then added kidney function and a representative inflammatory marker (WBC), followed by cardiometabolic comorbidities (12, 38). Two result patterns are noteworthy. First, robustness across sequential models—culminating in a fully adjusted OR of 1.61 (95% CI 1.20–2.16; *P* = 0.001)—suggests that the ePWV–dipstick proteinuria association remained statistically significant after adjustment for these covariates (Table 2). Second, tertile analysis indicated a stronger association in the highest ePWV stratum, and subgroup analysis revealed a stronger association in participants without dyslipidemia (interaction *P* = 0.001). Together, these design choices and findings position ePWV—computed from routine vitals, complementing recent large-scale reports of ePWV's association with adverse outcomes (12, 20).

### 4.5 Limitations

Several limitations of this study should be acknowledged. First, the cross-sectional design precludes causal inference, the observed association between ePWV and dipstick proteinuria may reflect reverse causality or unmeasured confounding. Second, the study was restricted to young adults aged 18–39 years, which limits the generalizability of our findings to older MASLD populations, in whom arterial stiffness is more heterogeneous and influenced by cumulative age-related and comorbidity-related changes. Third, the analysis was limited exclusively to participants with MASLD; no comparison group of non-MASLD individuals was included. Consequently, our findings can only be interpreted as an association observed within a selected MASLD population and cannot establish whether the ePWV-proteinuria relationship is specific to MASLD, stronger in MASLD, or similarly present among individuals without MASLD. Future studies with matched non-MASLD comparison groups are needed to determine whether MASLD status modifies the vascular-renal association. Fourth, while the ePWV equation used has been validated in general populations across a broad age spectrum (24), specific validation in Chinese young adults with MASLD is lacking, and our findings should be confirmed in other cohorts. Fifth, A major limitation is that alcohol-intake data were missing for 1,634 participants (99.6%) and therefore could not be incorporated into the analysis. Consequently, we were unable to quantitatively assess alcohol consumption or completely exclude potential misclassification related to alcohol exposure when defining MASLD. This limitation may have introduced residual misclassification and should be considered when interpreting the present findings. Future prospective studies with systematically collected alcohol-consumption data are warranted to validate these associations. Sixth, dipstick proteinuria, while practical for screening, is a semi-quantitative measure with known analytical limitations, including urine concentration, pH, hematuria, urinary tract infection, contamination, menstruation, exercise, fever, dehydration, and transient proteinuria (23), none of which were systematically available in our dataset, nor was confirmatory quantitative albuminuria testing performed. Furthermore, as ePWV is an estimated index rather than a direct measurement of arterial stiffness, and because MAP is a component of its calculation, the association may partially reflect the effect of blood pressure. Future studies incorporating directly measured cf-PWV and alternative approaches to account for the blood-pressure component of ePWV may help clarify the specificity of the observed association. Finally, the subgroup finding of a stronger association in participants without dyslipidemia was unexpected and warrants cautious interpretation. This may reflect residual confounding, differences in lipid-lowering treatment, multiple testing, or limited statistical power within subgroups.

## 5 Conclusions

Higher ePWV was associated with an increased likelihood of dipstick proteinuria among young adults with MASLD, and this association remained statistically significant after adjustment for demographic, anthropometric, renal-related, metabolic, and clinical factors. Restricted cubic spline analysis indicated a statistically significant nonlinear association across the observed ePWV range. The association varied according to dyslipidemia status, with a stronger estimated association among participants without dyslipidemia. Given the cross-sectional design, these findings should be interpreted as associations rather than evidence of causality. Further prospective studies are warranted to confirm these findings and determine whether ePWV is associated with urinary protein abnormalities and subsequent kidney outcomes among young adults with MASLD.

## Data Availability

The original contributions presented in the study are included in the article/Supplementary Material, further inquiries can be directed to the corresponding author.
