# Supplementary material for: Estimated pulse wave velocity and dipstick proteinuria in young adults with MASLD: a cross-sectional health-check analysis
Source: Front Med (Lausanne). 2026 Oct 7;13:1887276. doi: 10.3389/fmed.2026.1887276 (PMC13683076; doi:10.3389/fmed.2026.1887276)
Supplement: Supplementary file 1 [file Datasheet1.docx]

**Table S1. Assessment of multicollinearity among covariates.**

| **Variable** | **VIF** |
| --- | --- |
| Sex | 1.552 |
| Age | 1.293 |
| BMI | 3.081 |
| WC | 3.267 |
| SCr | 1.410 |
| BUN | 1.100 |
| Lp(a) | 1.024 |
| WBC | 1.143 |
| HTN | 1.041 |
| T2DM | 1.153 |
| Dyslipidemia | 1.095 |
| Urolithiasis | 1.022 |
| *H. pylori* infection | 1.068 |

Note: Multicollinearity was assessed using the variance inflation factor (VIF). A VIF <5 was considered to indicate no substantial multicollinearity. BMI, body mass index; WC, waist circumference; SCr, serum creatinine; BUN, blood urea nitrogen; Lp(a), lipoprotein(a); WBC, white blood cell count; HTN, hypertension; T2DM, type 2 diabetes mellitus; *H. pylori* infection, Helicobacter pylori infection.

**Table S2. Sensitivity analyses replacing WBC with individual inflammatory markers in the fully adjusted model**

|  | **Inflammatory marker** | **ePWV OR (95% CI)** | **T2 vs T1 OR (95% CI)** | **T3 vs T1 OR (95% CI)** | **P for trend** |
| --- | --- | --- | --- | --- | --- |
| Model A | NEU | 1.61 (1.20–2.15) | 1.65 (0.95–2.91) | 2.65 (1.54–4.68) | 0.002 |
| Model B | MON | 1.57 (1.17–2.11) | 1.55 (0.89–2.75) | 2.51 (1.45–4.44) | 0.003 |
| Model C | LYM | 1.63 (1.22–2.18) | 1.68 (0.97–2.96) | 2.71 (1.58–4.79) | 0.001 |
| Model D | CRP | 1.62 (1.21–2.17) | 1.66 (0.96–2.93) | 2.69 (1.57–4.74) | 0.001 |

**Note:** Four separate sensitivity models were fitted. In each model, WBC in Model 3 was replaced by one inflammatory marker (NEU, MON, LYM, or CRP), while all other covariates remained unchanged. NEU, MON, LYM, and CRP were not entered simultaneously. The common covariates in all sensitivity models were sex, age, BMI, WC, SCr, BUN, Lp(a), HTN, T2DM, dyslipidemia, urolithiasis, and H. pylori infection. All VIFs in the four sensitivity models were <5.

**Figure S1. Distribution of ePWV by dipstick proteinuria status.**

**Figure S2. Restricted cubic spline analysis of the association between ePWV and dipstick proteinuria.** The y-axis represents the adjusted predicted probability of dipstick proteinuria, with all other covariates in Model 3 held at their mean values (for continuous variables) or reference categories (for categorical variables). The solid line represents the predicted probability, and the shaded area indicates the 95% confidence interval. Four knots were placed at the 5th, 35th, 65th, and 95th percentiles of the ePWV distribution. P for nonlinearity = 0.009.
